# Coronary Cameral Fistula: A Rare Case Presenting With Non-ST-Segment Elevation Myocardial Infarction and Pulmonary Arterial Hypertension

**DOI:** 10.7759/cureus.61604

**Published:** 2024-06-03

**Authors:** Jimmy Saleh, Mersal Samimi, Asseel Al-Bayati, Henning Rasmussen, Richard Kiel

**Affiliations:** 1 Internal Medicine, University of California San Francisco, Fresno, Fresno, USA; 2 Cardiology, University of California San Francisco, Fresno, Fresno, USA

**Keywords:** congenital cardiac anomaly, cardiac chest pain, non-st segment elevation myocardial infarction (nstemi), – pulmonary hypertension, coronary cameral fistula

## Abstract

Coronary cameral fistulas (CCFs) are uncommon congenital or acquired anomalies characterized by abnormal connections between a coronary artery and a cardiac chamber. While often asymptomatic and incidentally detected, symptomatic presentations are rare, and symptoms may vary depending on the size and location of the fistula. We present the case of a 67-year-old female with complaints of intermittent typical cardiac chest pain and exertional dyspnea. Further evaluation revealed a CCF originating from the left anterior descending coronary artery and the left ventricle. Additionally, the patient was found to have pulmonary hypertension on right heart catheterization. This case highlights the importance of considering CCF in the differential diagnosis of chest pain, particularly in the presence of atypical symptoms and associated pulmonary hypertension (WHO Group 4). Further research is warranted to elucidate the optimal management strategies for symptomatic CCF, especially in cases complicated by pulmonary hypertension.

## Introduction

Coronary cameral fistulas (CCFs) are rare anomalous communications that occur between one or more coronary arteries and a cardiac chamber. This anomaly more frequently arises from the right coronary system (≈55%) but can originate from the left side (35%) or bilaterally (5%) [1]. These fistulas most commonly terminate on the right side of the heart (most frequently the right ventricle (41%), then the right atrium and pulmonary artery (PA), with only 3% terminating in the left ventricle (LV)) [1,2].

Most CCF patients are asymptomatic, while the most common presentation in symptomatic patients includes chest pain or heart failure; however, arrhythmias are rarely associated. A review study on coronary artery fistulas showed that 55% of patients were asymptomatic, 34% had angina, and 13% had heart failure [3-6]. Symptomatic fistulas can be managed with transcatheter embolization or surgical closure [7].

Below, we present a unique case of an elderly female presenting with ischemic chest discomfort with dynamic electrocardiogram changes along with pulmonary hypertension. Her coronary angiography revealed a rare left anterior descending (LAD) artery to the LV CCF.

## Case presentation

**Figure 1:**
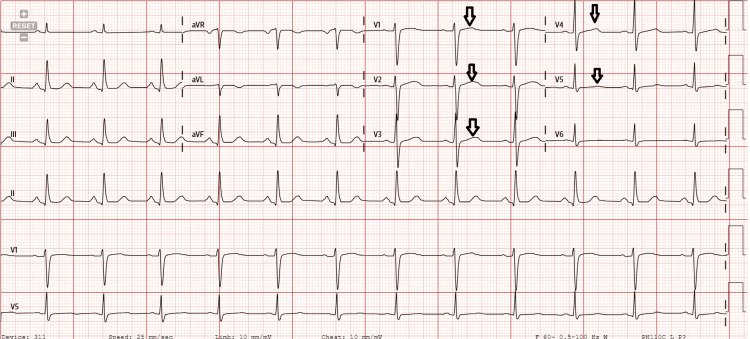
Prior electrocardiogram depicting a normal sinus rhythm pattern without T-wave inversions in V1-V5

**Figure 2:**
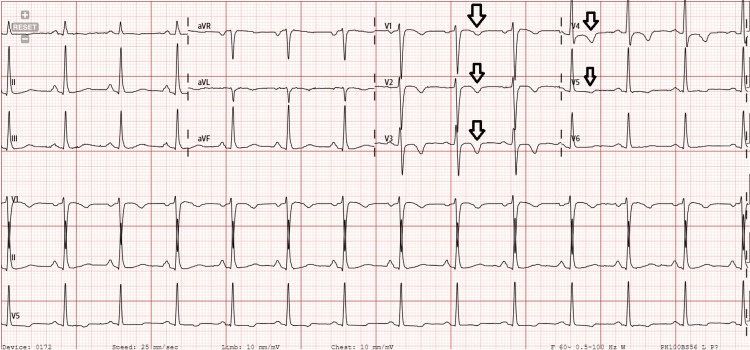
Electrocardiogram taken at the time of presentation with chest pain demonstrating T-wave inversions in leads V1-V5

A 67-year-old female with a past medical history of hypothyroidism, recurrent deep venous thrombosis, and pulmonary embolisms on enoxaparin 40 mg/daily and pulmonary hypertension (WHO Group 4) presented to the emergency department with exertional chest pain that was relieved with rest and described as a left-sided heaviness radiating to her left arm and left shoulder. The physical examination was unremarkable. Labs were notable for an initial troponin of 0.142 ng/mL (reference range ≤0.040 ng/mL) and an electrocardiogram with dynamic T-wave inversions (V1-5) not seen on previous EKGs (Figures 1, 2).

**Figure 3:**
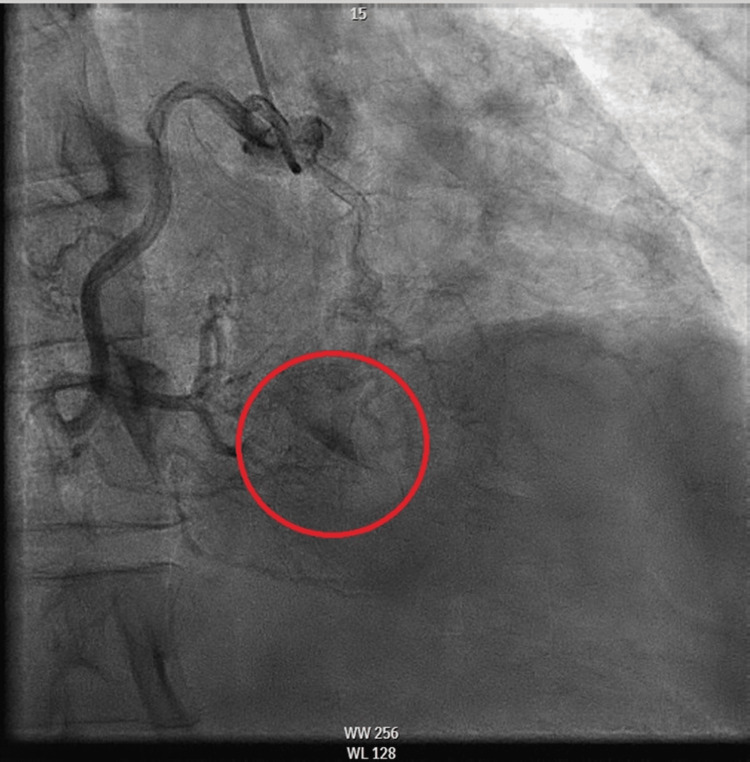
Angiographic photo displaying contrast injected into the RCA with evidence of contrast entering the LV LV, left ventricle; RCA, right coronary artery

**Figure 4:**
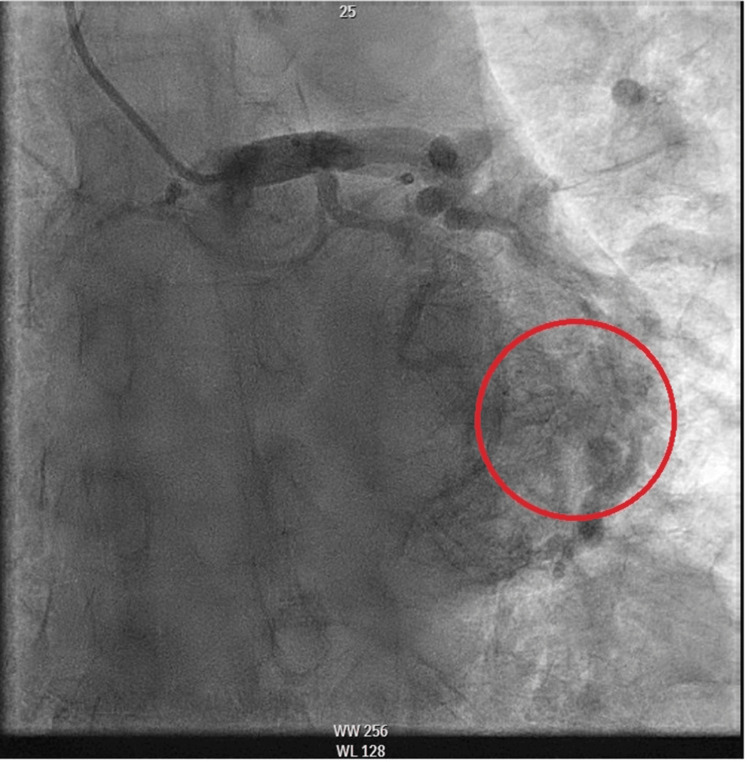
Angiographic photo showing a CCF originating from the LAD artery and terminating into the LV CCF, coronary cameral fistula; LAD, left anterior descending; LV, left ventricle

Given concerns for the ongoing acute coronary syndrome, the patient was put on aspirin loaded with 325 mg, started on a heparin drip (12 units/kg/hour), and given nitrates (5 mg/min) for continuous chest pain. A transthoracic echocardiogram showed normal right and left size and function; however, due to suboptimal Doppler imaging of the flow through the tricuspid valve, the estimation of PA systolic pressure using the maximal speed of tricuspid flow was unfeasible. The patient was taken to the cardiac catheterization lab for early invasive coronary angiography, which revealed mild compensated pre-capillary pulmonary hypertension and evidence of two CCFs, one originating from the right coronary artery (RCA) to the LV (Figure 3, Video 1) and the other from the LAD artery into the LV (Figure 4, Video 2).

**Video 1:** Angiography showing the cameral fistula from the distal RCA to the LV LV, left ventricle; RCA, right coronary artery

**Video 2:** Angiography revealing a CCF from the LAD artery into the LV CCF, coronary cameral fistula; LAD, left anterior descending; LV, left ventricle

The patient returned to the medical floor, where her symptoms were medically managed with a beta-blocker with significant improvement. The case was reviewed at the multidisciplinary heart team meeting for interventional cardiology and cardiothoracic surgery. The images were examined, and the literature was discussed. It was concluded that serial imaging with annual echocardiography and regular clinical review would be the best approach and that there was no current indication for invasive management. In addition to her chronic pulmonary embolisms likely causing her pulmonary hypertension, it was believed that her CCFs were further exacerbating her disease and causing unwanted symptoms.

## Discussion

CCFs are rare congenital or acquired anomalies that are often asymptomatic and incidentally detected. However, symptomatic CCFs, while rare, can occur, especially in cases of multiple fistulas. While consensus remains elusive regarding the management of symptomatic fistulas due to their rarity, various approaches, such as surgical repair, catheter closure, and medical management, have shown promise in trials. Focal fistulas with significant hemodynamic impact may benefit from closure procedures. Surgical closure has effectively addressed arterioluminal subtypes, while arteriosinusoidal subtypes often necessitate pharmacotherapy, particularly beta-blockers [8].

The majority of these fistulas arise from the RCA and terminate in the right side of the heart; however, on rarer occasions, they can originate from the LAD artery and seldomly terminate in the LV [2], as seen in our case. The symptoms associated with CCFs typically result from mechanisms such as the coronary steal phenomenon and diastolic overload, leading to manifestations like angina. Other complications include pulmonary hypertension as a result of sizeable fistulas leading to the shunting of blood from the coronary circulation to low-pressure pulmonary vascular beds [9]. The hemodynamic consequences of CCFs can vary, depending on their magnitude and the cardiac chamber or vascular site involved. Fistulas terminating into the right heart chambers may produce left-to-right shunts and volume overload of the pulmonary circulation, whereas fistulas to the left heart can cause left ventricular volume overload [10].

In summary, while asymptomatic CCFs are common and often incidentally detected, symptomatic cases, albeit rare, require careful consideration and management. Although advances in noninvasive imaging, including computed tomography coronary angiography, MRI, and transesophageal echocardiography, have improved diagnostic capabilities, coronary angiography remains the definitive diagnostic tool. The management of CCFs depends on the symptoms and size of the fistula. Treatment options for symptomatic patients with large fistulas include surgical ligation or percutaneous transcatheter closure. Although surgical obliteration of the fistula is the most effective treatment, both techniques lead to a good prognosis. It is important to continue antiplatelet therapy after the closure of the fistula. Beta-blocker use has been described in patients with fistulas not amenable to surgery and may be a great treatment option to control symptoms and reduce morbidity.

## Conclusions

CCFs are rare congenital anomalies that can present with a wide spectrum of symptoms, including pulmonary hypertension. Timely diagnosis and appropriate management, including surgical intervention when indicated, can lead to favorable outcomes and symptomatic relief in affected individuals. Early recognition and appropriate management are crucial to prevent unnecessary complications or adverse effects of medications assumed to target atherosclerotic diseases, hence affecting long-term outcomes.
